# Laparoscopy assisted abomasal cannulation in cadavers of bovine fetuses

**DOI:** 10.1186/s12917-022-03473-4

**Published:** 2022-10-25

**Authors:** Heytor Jales Gurgel, Francisco Décio de Oliveira Monteiro, João Pedro Monteiro Barroso, Loise Araújo de Sousa, Gabriela Melo Alves dos Santos, Kayan da Cunha Rossy, Verena Siqueira da Silva, Camila do Espirito Santo Fernandes, Carla Rozilene Guimarães Silva, Rodrigo dos Santos Albuquerque, Luisa Pucci Bueno Borges, Luiz Henrique Vilela Araújo, Daniele Lira dos Santos, Felipe Farias Pereira da Câmara Barros, Pedro Paulo Maia Teixeira

**Affiliations:** 1 Veterinary Medicine Institute of Pará Federal University, Belém, 68.740-970 Brazil; 2 Campus Araguatins of Federal Institute of Education, Science and Technology of Tocantins (IFTO), Palmas, 77.950-000 Brazil; 3 Veterinary Hospital, Veterinary Institute, Pará Federal University (HV/IMV/UFPA, Castanhal Campus II, Br 316, Km 62, Castanhal, PA 68743-97014.884-900 Brazil; 4 Rio de Janeiro Federal Rural University, Rio de Janeiro, 23890-000 Brazil

**Keywords:** Animal experimentation, Abomasum, Fistulation, Laparoscopy, Ruminants

## Abstract

**Background:**

Due to the complexity of ruminant digestion, cannulation of organs of the digestive tract has been carried out in order to advance the understanding of digestive physiology, nutrient degradability, gastrointestinal diseases and biotechnological research. The abomasal cannulation is interesting for nutritional studies, especially in suckling calves, to obtain fluid and abomasal content, evaluation of abomasal flow and function, and infusion of nutrients and drugs when it is intended to reach high concentrations in the organ. Conventionally, access and cannulation of digestive organs of ruminants has been performed by laparotomy, a method often criticized and classified as cruel by some sectors related to ethics and animal welfare. The aim of this present study is to describe and standardize a minimally invasive by laparoscopy assisted abomasal cannulation in bovine fetuses (cadavers), which had been previously slaughtered by accident and would be discarded in local slaughterhouses.

**Results:**

The abomasal cannulation technique was feasible, simple and did not present major difficulties. The surgical time for cannulation of the abomasum, from the insertion of the trocars to the completion of the technique with fixation of the organ to the abdominal wall, ranged from 9 to 27 min, with an average of 15.5 ± 6.62 min.

**Conclusions:**

The Laproscopic assisted abomasal cannulation in bovine fetuses was feasible and safe with minimal tissue injury to the abdominal wall and with short surgical time. More studies in the clinical routine related to minimally invasive abomasal content collection, abomasopexy and abomasotomy are required in order to demonstrate its impact and importance in bovine clinic.

## Background

The abomasum is the organ of the digestion of suckling calves essential for nutritional digestibility and animal performance, responsible for the enzymatic digestion of the milk-based diet [1]. Due to the complexity of ruminant digestion, cannulation of organs of the digestive tract has been carried out in order to advance the understanding of digestive physiology, nutrient degradability, gastrointestinal diseases and biotechnological research [2–4].

The abomasal cannulation is interesting for nutritional studies, to obtain fluid and abomasal content, evaluation of abomasal flow and function, and infusion of nutrients and drugs when it is intended to reach high concentrations in the organ [5]. Conventionally, access and cannulation of digestive organs of ruminants has been performed by laparotomy, and the ongoing search for new procedures aimed at reducing stress and post-surgical complications may allow for reliable data collection, after all, if the animal is stressed by pain or mishandling, the samples could be altered, and the experiment will be unsuccessful [4, 6–8].

The use of fetal cadavers from slaughtered cows is an important alternative method for refining research and obtaining skills and competences related to the development of a new surgical technique [9, 10]. The application of endosurgery in the cannulation of the abomasum must take into account that newborn calves have rumen reticulum and omasum in a rudimentary state, with intense enzymatic activity in the abomasum and intestine that shelter them to function as pseudo-monogastric animals [11].

The laparotomy for access, exteriorization and cannulation of the abomasum requires a surgical incision in the right flank and three-layer suture, and is therefore a more invasive and traumatic method when compared to minimally invasive procedures [12, 13]. Digestive organ cannulation using a minimally invasive technique can provide better patient recovery with fewer postoperative complications, improving animal welfare [13–15].

A laparoscopic abomasal cannulation technique described in sheep presented good performance and less pain induction in the animals [5, 13]. Another minimally invasive rumenostomy technique was efficient in sheep [15]. The laparoscopy was feasible for surgical resection of the umbilical vein and urachus of bovine fetuses in a previous study [9]. In this sense, minimally invasive procedures becomes increasingly relevant from the point of view of ethics in experimentation and animal welfare, as it allows for better postoperative and cosmetic results for the patient [13, 15].

Therefore, the aim of this present study is to describe and standardize a minimally invasive by laparoscopy assisted abomasal cannulation in bovine fetuses (cadavers), which had been previously slaughtered by accident and would be discarded in local slaughterhouses.

## Results

The bovine fetuses used in the research presented the abomasum with physical development and anatomical location according to the fetal ontogeny of the stomach of ruminants at this stage, making it possible to explore the visceral portion of the abomasum (greater curvature, lesser curvature and pyloric portion) with complete inspection and manipulation through clamping with laparoscopic babcock forceps. Post-mortem changes caused an excess of bloody fluid in the abdominal cavity of 2 fetuses, making it difficult to locate the abomasum.

**Fig. 1:**
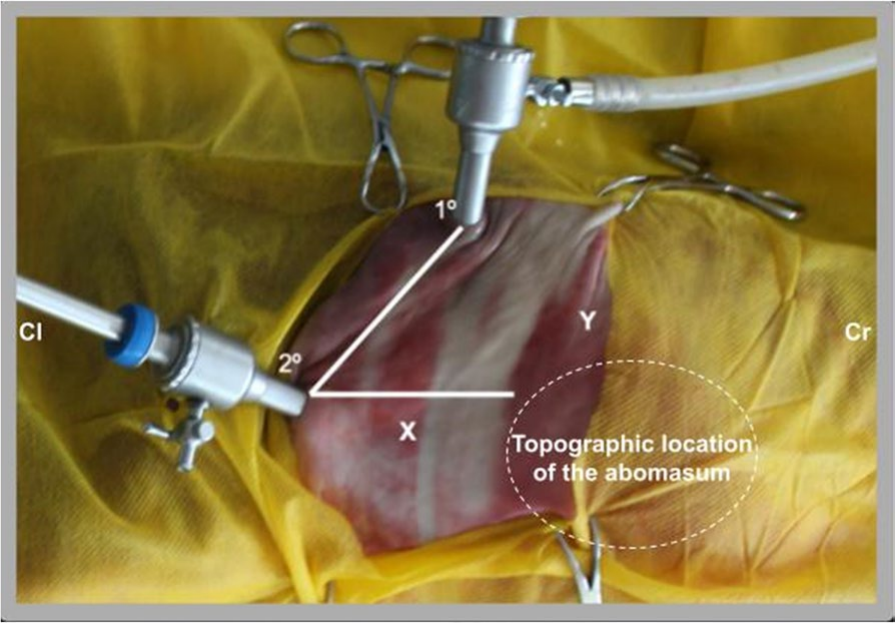
Video-assisted laparoscopic abomasostomy in bovine fetal cadavers. Arrangement of the portals on the right flank of a fetus in left lateral decubitus, first trocar through which the laparoscope is inserted and the CO2 hose is attached (1st), second trocar for insertion of the babcock forceps (2nd), right flank (x) and ribs (y). Cranial (Cr) and Caudal (Cr)

**Fig. 2:**
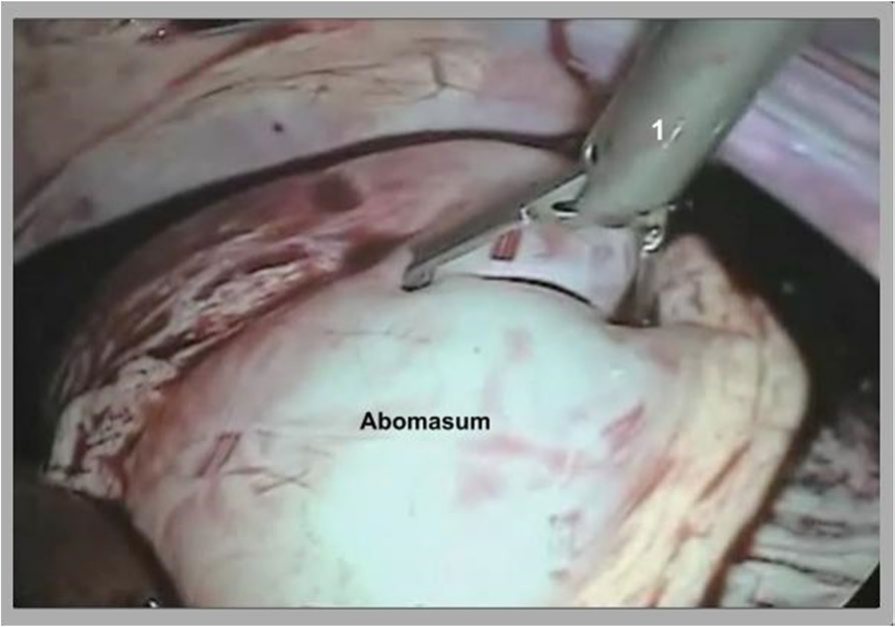
Intra-abdominal view with visualization of the abomasum and babcock forceps (1)

The approach in left lateral decubitus favored the intra-abdominal location of the abomasum due to its topographic location on the right, allowing quick visualization of the organ. The establishment of laparoscopic and working access portals in the right flank and right paralumbar fossa region, caudal to the last rib, obeying the triangulation between the portals and abomasum (Fig. 1), were essential for a better exploration of the abomasum of these fetuses, providing adequate visceral manipulation and excellent intra-abdominal visualization (Fig. 2).

**Fig. 3:**
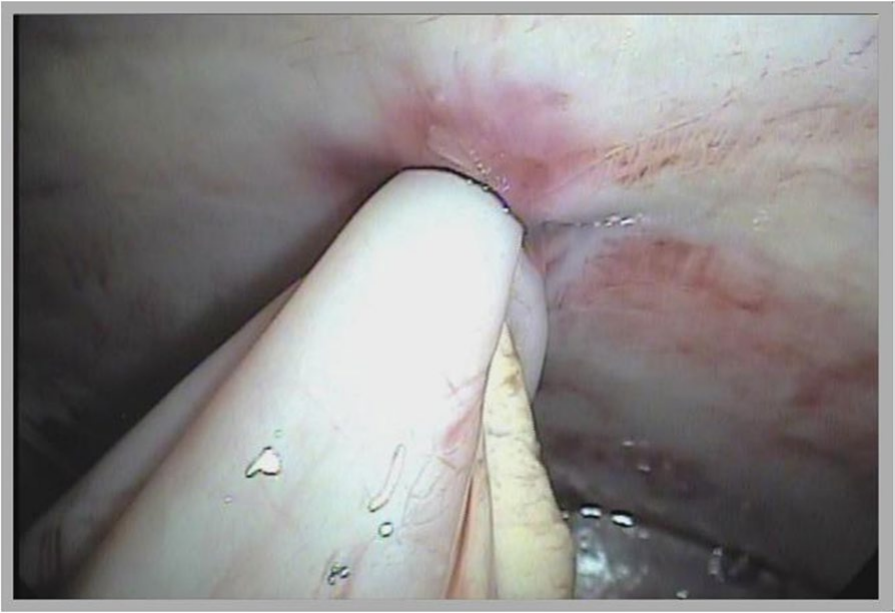
Intra-abdominal view of the abomasum after abomasostomy, fixation of the abomasal wall, region of greater curvature, to the abdominal wall

**Fig. 4:**
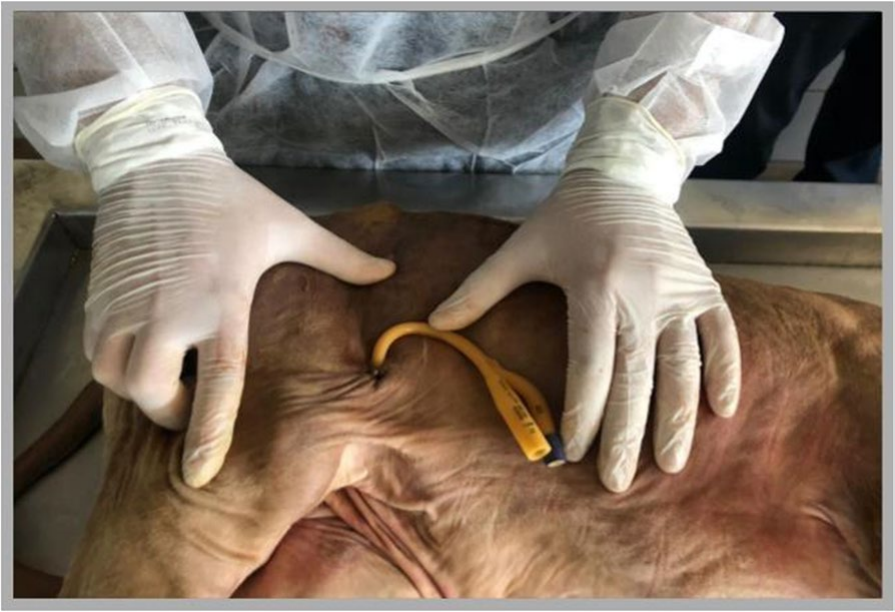
Final position of the Foley catheter at the end of the procedure

The position of the more ventral working port in the right flank region facilitated abomasopexy and abomosostomy with fixation of the foley tube in the abomasum (Fig. 3). The abomasal cannulation technique was feasible in the laparoscopic approaches performed (Fig. 4). After cannulation of the abomasum, it was possible to irrigate and drain liquid from the abomasum through the probe attached to a syringe, and it was found that there was no leakage in the abdominal cavity.

**Fig. 5:**
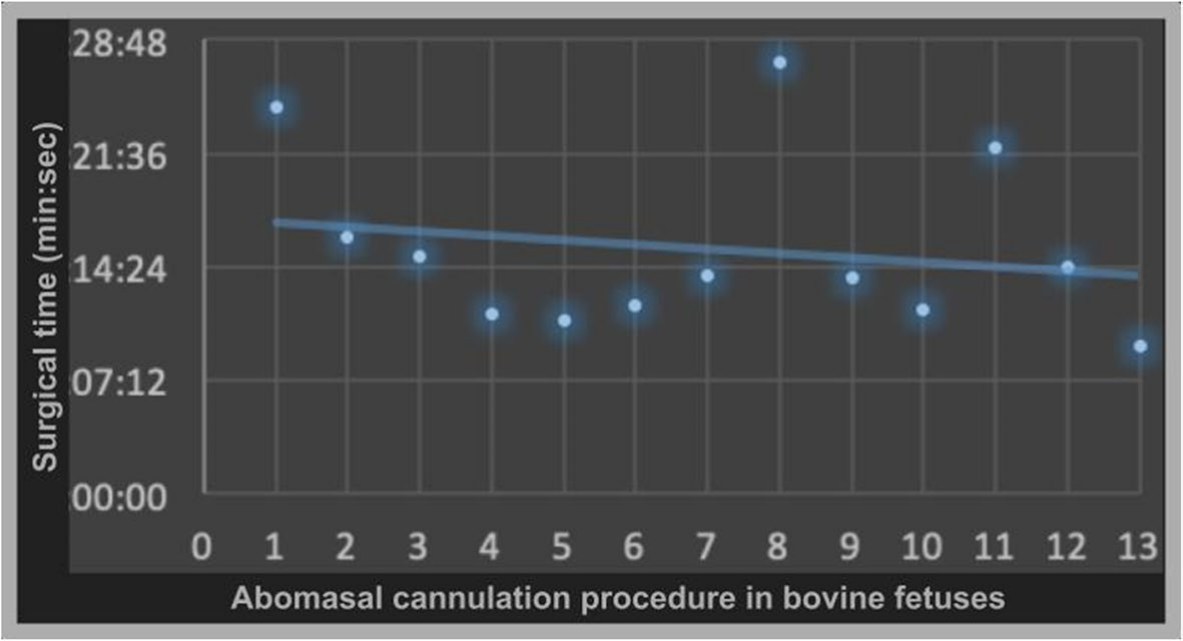
Scatter plot representing surgical time vs procedure in each fetus undergoing video-assisted laparoscopic abomasostomy, from the first to the last procedure. Blue line showing the tendency to reduce surgical time due to the learning curve

The surgical time for cannulation of the abomasum, from the insertion of the trocars to the completion of the technique with fixation of the organ to the abdominal wall, ranged from 9 to 27 min, with an average of 15.5±6.62 min. The surgical time data tended to decrease with each cannulation of the abomasum of the specimens submitted to the technique (Fig. 5).

## Discussion

Laparoscopy-assisted cannulation of the abomasum proved to be efficient and easy to perform. Minimally invasive cannulation was performed in sheep by Zhang et al (2016) and proved to be less traumatic than the conventional open technique. Thus, we hope that when performed on live animals, our technique can prevent further injuries to patients; less postoperative pain; and shorter surgical and anesthetic time.

The abomasum constitutes the largest gastric portion of newborn calves, as they need to use nutrients from their exclusively milk-based diet, not requiring as much of the rumen-reticulum and omasum, which are important for the fermentation of structural carbohydrates in roughages [1, 16]. In the laparoscopy of the fetuses, the abomasum was physically developed and located in the right half of the abdominal cavity, on the floor of the abdomen, where it was possible to explore and manipulate the organ and identify it for performing the laparoscopic cannulation technique.

The use of bovine fetal cadavers as an experimental model has already been described in other studies [9], as it facilitates training and adaptations of the technique without causing animal suffering or euthanasia [10]. Although the technique of abomasal cannulation by laparotomy has been described in live (animals) calves [2], the technique of the present study may prove to be a viable option for minimally invasive abomasal cannulation in live animals. This study should be complemented with alive calves, in order to demostrate its impact and importance in experimental and bovine clinic.

From an experimental point of view, minimally invasive cannulation of the abomasum may allow nutritional studies related to digestive physiology and nutrient degradability in the abomasum with better postoperative, aesthetic, ethical and animal welfare results [13, 15]. Once the abomasum is cannulated, it is possible to obtain abomasal content, evaluate the abomasal flow and function, in addition to infusing nutrients and drugs when high concentrations are intended to be reached within the organ [5]. Other clinical applications that can be tested are: biopsy to confirm the diagnosis of abomasal lymphosarcoma, therapy of perforating abomasal ulcers and recurrent abomasal impaction.The lateral approach from the right flank with the establishment of access portals were efficient, favoring the intracavitary handling [17]. Difficulties in manipulating the laparoscope and instruments were reported [5], but with an adequate planning for surgical accesses, a good visualization of the internal structures and their easy manipulation are achieved [9]. The establishment of laparoscopic portals on the right flank was chosen based on the topographical location of the abomasum, since access to the organ could be difficult from the left flank [18].

Laparoscopic abomasal cannulation through the ventral region of the abdomen is feasible, however, our technique of abomasal cannulation through the right flank may allow an easy handling of the cannula, due to its position in the flank, in addition to allowing future studies regarding the performance of this technique in animals in quadrupedal position This mean surgical time, which is shorter than the work performed on live animals, must change when the technique it is applied in vivo, as precautions must be taken to avoid spillage of abomasal fluid in the abdominal cavity [13]. The study can be a basic groundwork on further research on the pathophysiology and treatment of abomasal displacement in bovines [19].

The specimens did not yet have food content in the abomasum, however the tests allowed to introduce and collect content from the Foley catheter. This same type of cannula was introduced into the rumen of live sheep, allowing the collection of ruminal fluid, it also proves to be easy to replace in cases where the cannula has been removed spontaneously or by the animal [15]. Our results were similar to the results obtained by Santos et al. [15], who described a video-assisted laparoscopic rumenostomy technique in sheep, with an average surgical time of 13 minutes, with no significant difference in the time achieved in this experiment.

Abomasostomy with cannulation of the abomasum, both laparoscopic and conventional, were performed in sheep with a mean operative time longer than the mean operative time in our study (15.5 ±6.62 min), around 49 and 22 min, respectively [5, 13]. This mean surgical time, which is shorter than the work performed on live ruminants, must change when the technique it is applied in vivo, as precautions related to pre-surgical fasting, adequate abomasostomy and abomasopexy to avoid spilling abomasal fluid in the abdominal cavity.

A learning curve was observed, where the first abomasal cannulation procedure had a longer surgical time when compared to the subsequent procedures due to the initial adjustments of the technique and the skill of the surgical team, demonstrating a tendency of reducing the surgical time with the processing of the specimens. The first abomasostomy was the longest and the last was the shortest, however, there was little variation between them, demonstrating that this type of procedure has low complexity, without great difficulty to perform the procedure, which required minimal time to adapt and skill acquisition to the experimental technique.

The proposed abomasal cannulation proved to be feasible in bovine fetuses, and may constitute an alternative minimally invasive technique applied to calves and other ruminant species, requiring further studies related to the procedure. Another possibility of applying the technique, which also requires applied research, is its use in order to correct conditions related to displacement of the abomasum, considering that the resolution of this disorder is based on fixing the organ in the abdominal wall and controlling the amount of gases present in it [19].

## Conclusion

The laparoscopic assisted abomasal cannulation in bovine fetuses was feasible and safe with minimal tissue injury to the abdominal wall and with short surgical time. More studies in the clinical routine are required in order to demonstrate its impact and importance in bovine clinic.

## Methods

This study was carried out in accordance with the recommendations of the National Council for Experimentation Control in Brazil (CONCEA). This research was approved by the Animal Ethics and Welfare Committee of the Federal University of Pará (protocol N ° 4848261017). As the study corresponds to a new experimental technique, all surgical procedures were performed on cadavers from a local slaughterhouse with state industrial and sanitary certification. Thus, the procedures did not cause pain or suffering in animals, as they were performed on bovine fetuses procured from slaughtered pregnant cows. The experiment was conducted at the Institute of Veterinary Medicine (IMV) in Campus II of the Federal University of Pará (UFPA), located in the municipality of Castanhal, Pará, Brazil, and involved video-assisted cannulation of the abomasum in 13 cadavers (weighing between 30 and 40 kg, bovine fetuses from cows slaughtered in the last trimester of pregnancy). All steps of the procedure were performed by the same surgeon in a standard way.

The feasibility of abomasal cannulation and the surgical time were analyzed in all cadavers, being evaluated the feasibility of the technique and the surgical time.

### Instruments and equipment used in the study

The experimental simulation of the surgical procedures took into consideration all the surgical principles applicable to laparoscopy and the necessary equipment and instruments were used to perform the techniques. We used a 10-mm laparoscope, 10-mm or 5-mm Babcock forceps, 5-mm laparoscopic scissors, a set of gas insufflator/light source/monitor, and basic surgical instruments for conventional surgery.

### Laparoscopy assisted abomasal cannulation with two access ports

The anatomical specimens were placed in the left lateral recumbency position and underwent laparoscopy using two laparoscopic access ports in the right flank, with two 10-mm cannulas in the first and second access port.

The access ports were established in the right flank near the paralumbar fossa, caudal to the ribs, using the open technique [20]. Skin incisions of approximately 8 to 10 mm were made using a scalpel to insert the trocars transmurally into the abdominal cavity.

The first 10-mm laparoscopic access port with an insufflation valve was inserted, through which a carbon dioxide (CO2)-induced pneumoperitoneum of 8 mmHg was established, and the abdominal cavity was inspected by viewing the image on the monitor.

With adequate visualization of the abdominal cavity and, mainly, of the abomasum, the second trocar was inserted in a video-assisted way, caudally to the first trocar.

Using a Babcock atraumatic forceps, the abomasum was seized and suspended until it reached the second portal. Upon arriving on the portal way, the abomasum was held by 2 conventional Allis forceps. Thus, a puncture-incision was made on the wall of the abomasum with a scalpel. Through this incision, a Foley Catheter (2 mL, 2-Way, 22 Fr/Ch, 6.0 mm) was surgically inserted in the abomasal wall with two double concentric purse-string sutures in the abdominal wall. After the cuff was inflated, a purse-string suture was performed around the catheter, penetrating only the serous layer of the abomasum. The muscle layer was occluded with interrupted X-suture next to the catheter and the skin with interrupted U-suture, for synthesis of the surgical incision for the first access. Finally, the abdomen was deflated, the laparoscope and first trocar inserted were removed, the muscular layer and skin was closed the same as before. All sutures were performed using 2-0 nylon.

The cannula was tested after the end of the procedure with a 60 mL syringe containing saline solution, allowing irrigation and drainage from the interior of the abomasum. Laparoscopic intra-abdominal visualization was performed during the testing phase in order to verify possible leakage in the abdominal cavity. 

### Statistical analysis

The Shapiro–Wilk test was used to confirm that surgical time data were distributed normally. Descriptive statistics were processed using the BioEstat program, version 5.3. The confidence interval was 0.95, and when *p* ≤ 0.05, the difference was considered significant.

## Data Availability

All data generated or analysed during this study are included in this published article [and its supplementary information files].
